# The mechanism of mTOR (mammalian target of rapamycin) in a mouse model of polycystic ovary syndrome (PCOS)

**DOI:** 10.1186/1757-2215-5-38

**Published:** 2012-11-27

**Authors:** Aylin Yaba, Necdet Demir

**Affiliations:** 1 Department of Histology and Embryology, Istanbul Bilim University Faculty of Medicine, Istanbul 34394, Turkey; 2 Department of Histology and Embryology, Akdeniz University Faculty of Medicine, Antalya 07070, Turkey

**Keywords:** mTOR, PCOS, Mouse, Ovary

## Abstract

Polycystic ovary syndrome (PCOS) is a common and complex endocrine disorder affecting 5-10% of women in reproductive age that is characterized by hyperandrogenism, oligo- or anovulation and infertility. However the pathophysiology of PCOS still remains unknown. The mammalian target of rapamycin (mTOR) is a central component that regulates various processes including cell growth, proliferation, metabolism, and angiogenesis. mTOR signaling cascade has recently been examined in ovarian follicles where it regulates granulosa cell proliferation and differentiation. mTOR functions as two complexes, mTOR complex 1 and 2. Therefore, we hypothesized that mTORC1 and/or 2 may have important role in proliferation of theca and granulosa cells in PCOS. In the present study, we sought to determine the mTOR signaling pathway in PCOS mouse ovary. We designed 3 groups: Control (C, no treatment), PCOS (P, The injection of DHEA (6 mg/100 g BW in 0.1 ml of sesame oil) (s.c) for 20 consecutive days), Vehicle (V, daily (s.c) sesame oil alone injection). Our results showed that mTORC1 and mTORC2-mediated signaling may play a role in PCOS mouse ovary. These findings provide evidence that mTORC1 and mTORC2 may have responsibility in increased ovarian follicular cell proliferation and growth in PCOS. Consequently, these results suggest that the mTOR signaling pathways (mTORC1 and mTORC 2) may create new clinical strategies to optimize developmental competence of PCOS should target correction of the entire follicle growth, oocyte development process and anovulatory infertility in PCOS.

## Introduction

Polycystic ovarian syndrome (PCOS) a complex, multifactorial endocrinopathy which affects approximately 5 to 10% of reproductive-aged women, is the most common cause of anovulatory infertility. Because it is a highly heterogeneous syndrome with a variable clinical presentation, criteria for diagnosis have been debated. The disease begins at menarche, and symptoms generally include oligomenhorrhea, amenorrhea, anovulation, numerous antral follicles, hypersecretion of circulating LH but with lower or equivalent FSH levels, obesity, hirsutism, and insulin resistance [1]. Additionally, PCOS include hypothalamic-pituitary dysynchrony, aberrant gonadotropin pulsatile secretion, granulosa/theca cell dysfunction, and various metabolic derangements including exaggerated ovarian androgen production, hyperinsulinemia, and insulin resistance [2-7]. The antral follicles produce high levels of androstenedione, testosterone, and 17αOH-progesterone. The cysts themselves are remnants of atretic follicles, fluid filled and devoid of granulosa cells. Polycystic ovary syndrome (PCOS) is the most common cause of anovulatory infertility [8]. Although the mechanism of anovulation remains uncertain, it is known that genetic and environmental factors play a role in the origin and development of this disorder [9-11]. The endocrine manifestations of PCOS include increased androgen production of ovarian and/or adrenal origin and arrested follicular development leading to chronic oligo- or anovulation [9]. Many authorities utilize the guidelines of Rotterdam/ASRM-sponsored PCOS Consensus Workshop Group [12] and require the presence of at least two of the following: oligoovulation and/or anovulation, evidence of clinical or biochemical hyperandrogenism, and the presence of polycystic ovarian morphology during ultrasound examination.

The disrupted hypothalamic-pituitary synchrony, increased gonadotropin pulsatile secretion, destroyed oocyte-granulosa cell interaction, increased ovarian androgen production, hyperinsulinemia, and insulin resistance included the etiologies of PCOS [13]. Studies confirmed that the DHEA-treated PCOS murine model exhibits many of the salient features of human PCOS [14,15]. The dehydroepiandrosterone (DHEA) is one of the most abundant circulating androgens in women with PCOS. Histological examination of ovaries from the DHEA-treated mice reveal different size of preantral and antral follicles with a thickened layer of hyperfunctional theca cells and a compacted formation of granulosa cells in the ovarian cortex. Additionally, DHEA-treated mice show increased serum estradiol and progesterone levels because of the high level circulating androgens [16]. The objective of this study was to determine the effects of androstenedione on mTOR signal during ovarian follicular growth and development. Thus, the present study was designed to study the possible role of mTOR complexes in PCOS mouse model. In order to determine how the functionality of ovarian tissue was modified with cystogenesis, the endocrine markers serum progesterone (P) and estradiol (E) were evaluated.

The mammalian target of rapamycin (TOR) gene product is a ubiquitous serine/threonine kinase that has been implicated in the control of different stressors, growth factors, nutrients, and hormones, which participates in the control of key cellular functions, including cell proliferation, growth, and metabolism [17-21]. mTOR forms two functionally distinct multiprotein complexes, mTORC1 and mTORC2, each of which has defined roles in the control of cell growth and fate [17,22]. mTORC1, which is rapamycin-sensitive complex, is composed of mTOR, Raptor, and mLST8 (also called GβL). Additionally, mTORC1 associates with PRAS40 (proline-rich Akt/protein kinase B (PKB)), FK506-binding protein 38 (FKBP38) and Rag GTPases. mTORC2, which is rapamycin-insensitive and growth-factor-responsive, consists of mTOR, Rictor, Sin1, mLST8, and PRR5/PRR5L (Protor 1/Protor 2). PRR5 is not required for the interaction between mTOR, Rictor, Sin1, and mLST8 [23-25]. mTORC1 and mTORC2 signal via distinct pathways to control a wide variety of cellular processes. These mTOR-regulated processes mediate the accumulation of cellular mass and thereby ultimately determine cell size. The processes controlled by mTOR include, but are not limited to, translation, ribosome biogenesis, nutrient transport, autophagy and AGC (cAMP-dependent protein kinase (PK**A**)/protein kinase G (PK**G**)/protein kinase C (PK**C**)) kinase activation.

mTOR signaling has been shown to have important role in the control of puberty onset, gonadotropin secretion, and they showed rapamycin treatment decrease LH secretion in the female rats [26]. We previously demonstrated that mTOR acts as a novel mitotic survival checkpoint to regulate follicle growth in vivo [27]. Therefore, we suggested that mTOR may have responsible excess granulosa and theca cell proliferation and hormonal dysfunction in PCOS. Here, we aimed to determine mTOR signal proteins in DHEA-treated PCOS mouse model. Thus, we have chosen to start DHEA treatment of mice at the prepubertal age of 25 days in order to investigate the mTOR signal proteins in the present study.

## Materials and methods

### Animals

The studies included in this work were performed in accordance with the Akdeniz University Institutional Animal Care and Use Committee Policies for Animal Use under an approved animal protocol. Handling and euthanasia of mice were performed per the Akdeniz University Institutes of Health Guide for the Care and Use of Laboratory Animals. The hyperandrogenized environment of PCOS was reproduced in mice by injection of DHEA [16,28,29]. Briefly, 15 female prepuberal (25 days old) mice of the Balb/C strain were daily injected (sc) with DHEA (IL,USA) (6 mg/100 g body weight, dissolved in 0.01 ml 95% ethanol [30,31] and mixed with 0.09 ml sesame oil) for 20 consecutive days (DHEA-treated PCOS group, (P)). The control group consisted of 15 mice injected with 0.09 ml sesame oil and 0.01 ml 95% ethanol daily for 20 consecutive days (oil group (vehicle (V))) and 15 untreated mice (control group (C)). All of these mice were raised and housed under controlled temperature (22°C) and illumination (14 h light: 10 h dark; lights on at 05:00 hours). After 20 days of treatment, animals were killed using cervical dislocation after ketamine anesthesia. After their removal, the ovaries were immediately fixed with fresh 4% paraformaldehyde for 6 hours for immunostaining.

### Morphological studies

To study the effect of DHEA on cyst formation, ten ovaries from the control group and ten from DHEA-treated, and 10 from vehicle group, fixed as described above, were embedded and samples were cut into 5 μm sections and deparaffinized in xylene and rehydration in a graded series of ethanol's. Afterwards sections were stained with hematoxylin and eosin (DAKO Corporation, Carpinteria, CA, USA) for histological analysis of cyst formation.

#### Hormone Assay

Blood for hormonal determinations was obtained by cardiac puncture from mice treated with DHEA and in proestrous and estrous control mice. After centrifugation, plasma was stored at -20°C until assayed. The levels of 17β-estradiol and progesterone were determined for each plasma sample. Serum Estrogen (ERK R7005, Endocrine Technologies) and Progesterone (ERK R7011, Endocrine Technologies) levels were determined by ELISA. We used μQuant Scanning Microplate Spectrophotometer 450 nm wave length.

#### Immunostaining for mTOR and P-mTOR

mTOR and P-mTOR protein expression detected in paraffin sections. Paraffin-embedded samples were cut into 5 μm sections were collected on poly-L-lysine coated slides (Sigma, St. Louis, MO, USA) and incubated overnight at 56°C. Tissue sections were deparaffinized in xylene and rehydration in a graded series of ethanol’s, antigen retrieval was performed by microwaving in EDTA (pH: 8.0). For colorimetric detection of mTOR and P-mTOR, sections were immersed in 3% hydrogen peroxide in methanol for 10 minutes to block endogenous peroxidase activity. Slides were then incubated in a humidified chamber with TBS-T (Tris-buffered saline containing 0.1% Tween-20 and 5% normal goat serum; Sigma, St Louis, MO) for 1 hour at room temperature. After removing excess blocking solution, the sections were incubated with rabbit anti-mouse mTOR (1:100 dilution; Cell Signaling Technology, Danvers, MA) overnight in a humidified chamber at 4°C. The sections were washed 3 times for 5 minutes each with TBS-T and then incubated in biotinylated anti-rabbit secondary antibody. Incubation in streptavidin–horseradish peroxidase (HRP) conjugates (ABC Elite HRP staining kit; Vector Laboratories, Burlingame, CA) followed for 30 minutes. After washing, the sections were incubated in DAB (Sigma).

For double staining with P-mTOR, after developing mTOR immunoreaction with DAB, the sections were re-incubated with primary antibody against P-mTOR overnight in a humidified chamber at 4°C. Afterwards, the sections were incubated with a biotinylated secondary antibody (K0676; Dako), followed by a streptavidin-alkaline phosphatase complex (K0676; Dako) for 20 min each. Slides were developed in Fast Red chromogen (Dako Fast Red Substrate System; K0699; Dako) counterstained with Mayer’s Hematoxylin (S3309; Dako). For controls, sections were treated with appropriate mouse IgG diluted to the same final protein concentration as the primary antibody. Photomicrographs were taken with an Axioplan microscope (Zeiss, Oberkochen, Germany).

#### Western blot analysis

Total protein from the ovary tissues (n = 10) from each group were extracted using T-PER tissue protein extraction reagent (Pierce, Rockford, IL, USA), supplemented with protease inhibitor cocktail (1 mM Na3VO4, 10 μg/ml leupeptin, 10 μg/ml aprotinin and 1 mM phenylmethylsulphonylfluoride; Calbiochem, San Diego, CA, USA). The protein concentration was determined by Bio-Rad Protein Assay (Bio-Rad Laboratories, Hercules, CA, USA). Western blot analysis was performed as described previously [32]. Briefly, 20-μg of protein was loaded into each lane, separated electrophoretically by SDS–PAGE using 7.5% Tris–HCl gels, and electroblotted onto nitrocellulose membrane (Bio-Rad Laboratories). The membrane was blocked with 5% non-fat dry milk in TBS-T buffer (0.1% Tween-20 in Tris-buffered saline) for 1 h to reduce the non-specific binding. The membrane was then incubated with rabbit polyclonal mTOR antibody (1:1000 dilution; Cell Signaling Technology, Danvers, MA, USA) overnight at 4°C, and washed three times with TBS-T for 20 min. Then, the membrane was incubated for 1 h with peroxidase-labeled goat anti-rabbit IgG (Pierce; USA) and subsequently washed with TBS-T three times for 20 min. mTOR protein expression was detected using chemiluminescence detecting reagents (Perkin Elmer Life Sciences, Boston, MA, USA) and exposure of the membrane to BioMax film (Kodak, Rochester, NY, USA).

We repeated same procedure for P-mTOR (Serin-2448), P-mTOR (Serin-2481), Raptor, Rictor, P70S6K, Phospho-P70S6K, PKCα, Phospho–PKCα and SET8 (PCNA). We used β-actin for internal control.

#### RT-PCR

For reverse transcription polymerase chain reaction (RT-PCR), RNA was extracted from whole ovaries as follows (n = 10). Total ovary tissue homogenized in 1 ml of Trizol Reagent (Invitrogen), followed by RNA extraction. Reverse transcription was performed upon 1 μg of total RNA using Superscript II Reverse Transcriptase according to the manufacturer’s protocol. PCR reaction was carried out in 25-μl reaction. PCR was performed using 2 μl of each RT sample within a reaction mix containing 1X Buffer D, 10 pmol of each primer, and Taq Polymerase. All PCR reactions were performed using a hot start at 95°C for 5 minutes. 35 PCR cycles were performed for all target genes and control β-actin. Primers used for the amplification of gene mTOR, Raptor, Rictor, LST8 (GβL), P70S6K and PKCalpha were as follows (oligonucleotide sequences listed 5’ to 3’ orientation and product size follow gene name). All primers were designed such that a genomic exon was spanned, and mock reversed-transcribed control samples were run versus every experimental sample (Table 1).

**Table 1:** Primers list

mTOR	150 bp	forward, TTG GAG TGG CTG GGT GCT GA
		reverse, AAG GGC TGA ACT TGC TGG AA
Raptor	148 bp	forward, GCC ATC ACAGAT ACC ATC GC
		reverse, CTG CTT ACT GGGGTG CAG TT
Rictor	110 bp	forward, GAG AAC GTC CCG CTC GAT CT
		reverse, TGG CCCAGC TTT CTC ATA TT
LST8 (GβL)	138 bp	forward, GAC TAA GGC AGA GTG CAG AG
		reverse, AAA AGC GCA CCG TGT GGT CA
P70S6K	300 bp	forward, CTTGGCGAAT TAAGGGCTGC
		reverse, GCATAGGCCAGTTCTACAAT
PKCalpha	300 bp	forward, GTCCTGCACCGGTTGGCGAA
		reverse, GACCCACAGTGATCACAGAA
β-actin	399 bp	forward, GAT GAC GAT ATC GCT GCG CTG
		reverse, GTA CGA CCA GAG GCA TAC AGG

### Statistical analysis

Groups were compared by one-way ANOVA followed by *post hoc* Holm-Sidak test. Statistical calculations were performed using Sigma Stat for Windows, version 3.0 (Jandel Scientific Corp., San Rafael, CA). P < 0.05 was considered statistically significant.

## Results

### Morphological evaluation

#### Light microscopy

**Figure 1:**
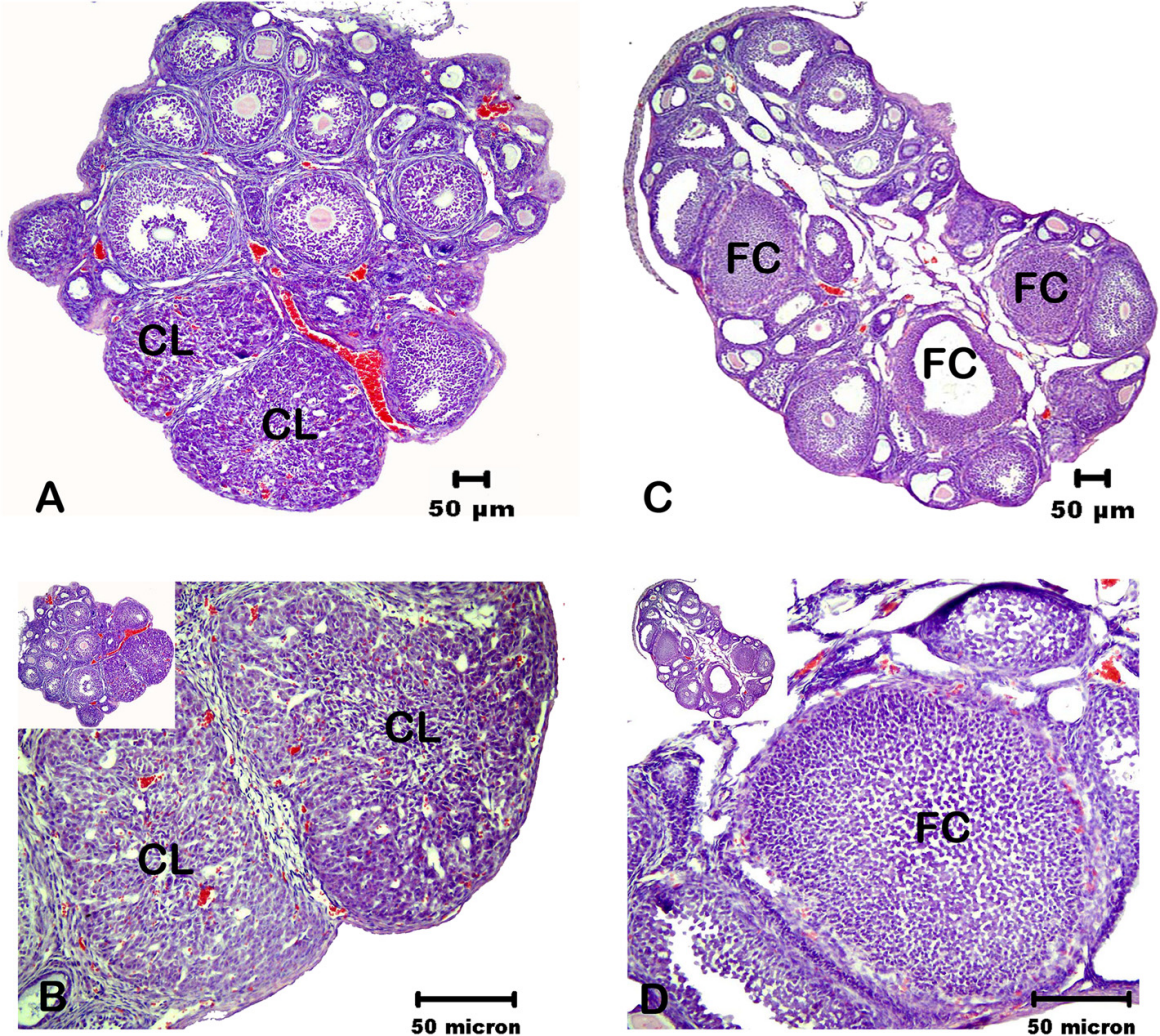
**Morphological comparison of control and DHEA-treated PCOS mouse ovary.A**, control ovary; **B**, Corpus Luteum (CL) structure from control ovary. **C**, DHEA-treated PCOS mouse ovary; showed different stage developing follicles but an increase in the number of cysts, preantral follicle and atretic follicles. **D**, Follicular cyst (FC) structure from DHEA-treated PCOS mouse ovary. The morphology of cysts is characterized by a thin layer of theca cells and a compacted formation of granulosa cells.

The general appearance of the control group ovary resembles normal histology and a peripheral cortex containing large numbers of follicles in various stages of development. We observed follicular developmental synchronization and compact stromal tissue between the developing follicles (Figure 1A). Oocytes in the follicles present normal healthy morphology. Medullar region shows normal vessels network areas in the center of ovary. We also detected corpus luteum (CL) structure in the control group ovary (Figure 1A and B).

**Figure 2:**
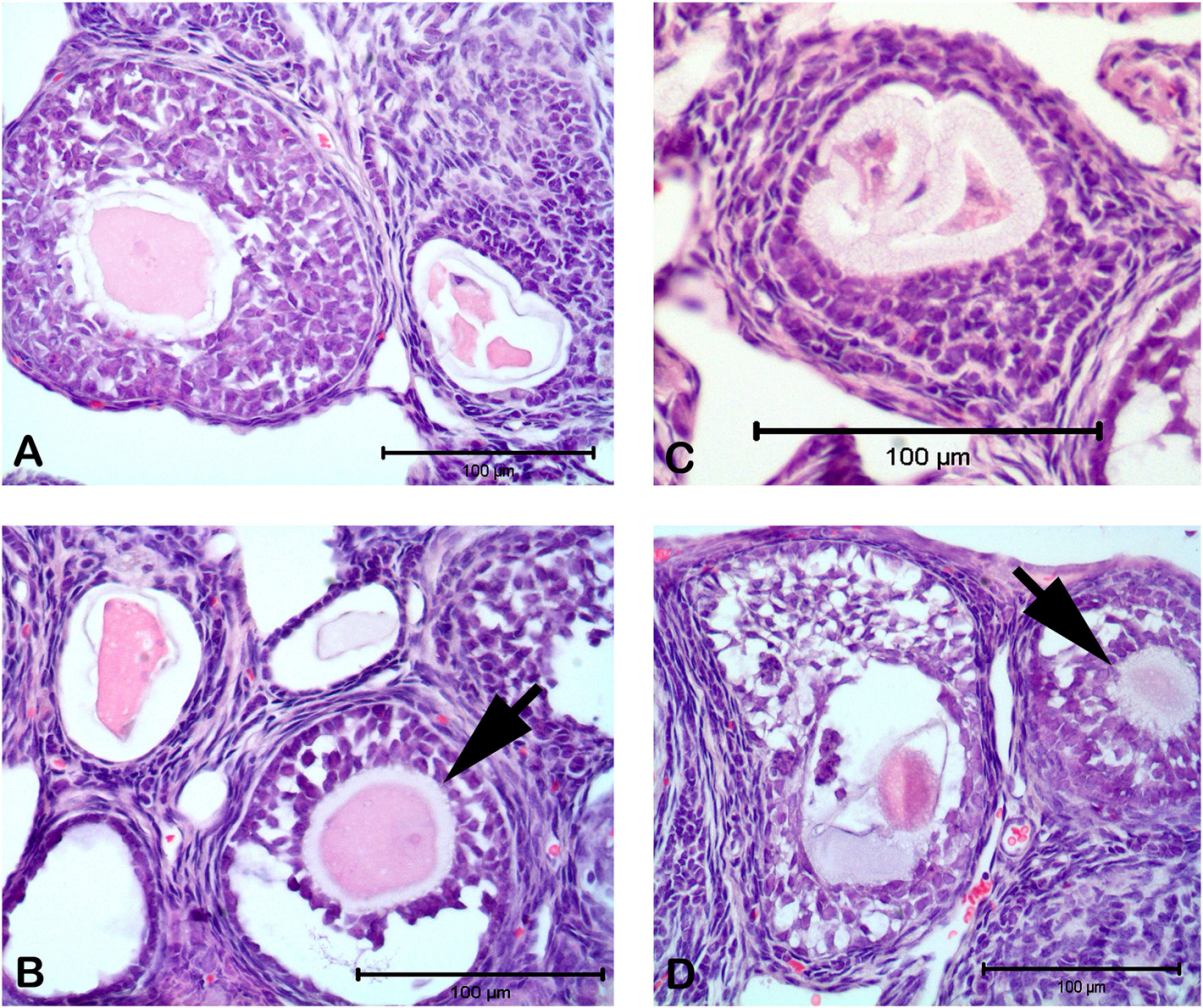
**Atretic follicles from different stage of follicular development in DHEA-treated PCOS mouse ovary.** Unhealthy oocytes have seen with degenerated cytoplasm and zona pellucida (**A**–**D**). The arrows show early preantral follicles with compact oocytes and zona pellucida (Figure B and D).

Histological examination the cortex of ovaries from DHEA-treated mice showed different stage developing follicles but an increase in the number of antral (Figure 1C and D) and preantral follicles (Figure 1C). We observed degenerated granulosa cells and oocytes in atretic follicles (Figure 2A–D). We detected unhealthy primary oocyte morphology in PCOS group. Histological analyses revealed the absence of corpora lutea (CL) in PCOS ovary (Figure 1C and D), which showed also abundant antral follicle and small atretic follicles, in contrast with the presence of abundant corpora lutea and large antral follicles in control ovaries. Ovulation does not occur in DHEA-treated group and some of follicles enter atretic process and oocytes in the follicle degenerated. Additionally, we detected characteristic theca cell luteinization (Figure 1C and D).

PCOS ovaries were larger than control and vehicle groups because of numerous antral follicles. Stromal species between follicles were incompact in the cortex layer of ovary. We observed increased medullar area and enlarged vessels network. Vehicle group ovary represents same morphological findings with control ovary.

#### Hormone assay

**Figure 3:**
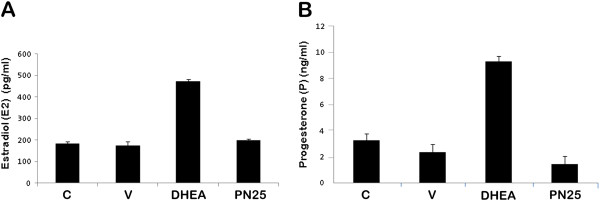
**Serum Estradiol (E2) (A) and Progesterone (P) (B) levels were evaluated by ELISA.** Female mice of 25 day old (before treatment postnatal 25-day old, PN25), 45 day old (25 day old BalbC mice + 20 day treatment (DHEA-treated/sesame oil-treated) or no treatment (control group) = 45 day old). The levels of estradiol and progesterone examined increased after treatment with DHEA. C: Control group, DHEA: DHEA-treated PCOS group, V: Vehicle group.

Female mice of 25 day old (Postnatal 25 (PN25), before treatment), 45 day old (25 day old BalbC mice + 20 day treatment (DHEA-treated/sesame oil-treated) or no treatment (control group) = 45 day old) were sacrificed at the proestrus stage (based on vaginal smears) in order to measure gonadotropin levels during the follicular growth phase, but not the ovulation phase. As a measurement of ovarian function in the cyst pathology, serum Estradiol (E2) and Progesterone (P) levels were evaluated by ELISA (Figure 3A and B, respectively). The levels of steroid hormones determined in DHEA-treated and control animals. The levels of estrogen and progesterone examined increased after treatment with DHEA (Figure 3A and B).

#### Immunohistochemistry

**Figure 4:**
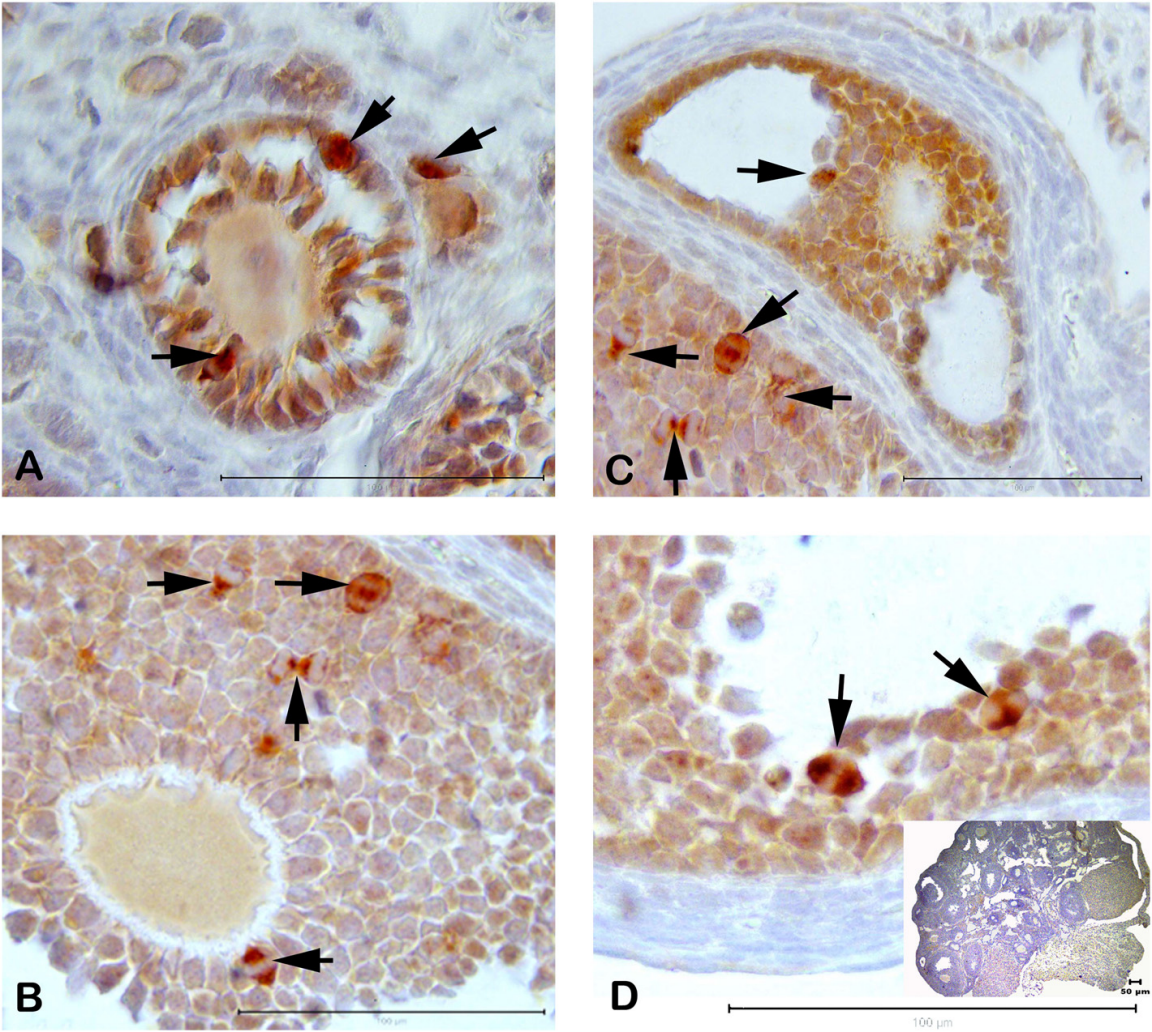
**Expression of mTOR and serine 2448–phosphorylated form of mTOR (P-mTOR) double staining by immunohistochemistry in DHEA-treated mouse ovary.** mTOR expression was shown mainly cytoplasmic nature of total within granulosa cells in various stages of follicular development in control, DHEA-treated and vehicle groups. P-mTOR expression was highly enriched in mitotic granulosa cells in all stage of developing follicles in three groups (arrow, **A**–**D**). Negative control has no staining (**D**, insert).

Expression of mTOR and serine 2448–phosphorylated form of mTOR (P-mTOR) was assessed by immunohistochemistry (Figure 4A–D). Generally, mTOR expression was shown mainly cytoplasmic nature of total within granulosa cells in various stages of follicular development in control, DHEA-treated and vehicle group. P-mTOR expression was highly enriched in mitotic granulosa cells in all stage of developing follicles in three groups. When we elevated all groups for mTOR staining pattern, we did not detect any difference but P-mTOR showed more positive staining in mitotic granulosa cells than control and vehicle groups. There was no difference for granulosa cells between control and vehicle groups for mTOR and P-mTOR immunostaining. We also detected cytoplasmic mTOR immunostaining in primary and secondary oocytes in different stage of developing follicles but not P-mTOR (Figure 4A–D). Granulosa lutein cells showed moderate mTOR staining in the control group ovary. There is no mTOR and P-mTOR immunoreactivity in negative control slides (Figure 4D, insert).

#### Western blot

mTOR is associated with two distinct complexes, mTORC1 and mTORC2. mTORC1 is rapamycin-sensitive and promotes cell growth and autophagy largely through activation of P70S6K1 and through phosphorylation P-P70S6K1 (by phosphorylation of P70S6K1threonine 389). mTORC2 is rapamycin-insensitive and regulates cytoskeletal dynamics and actin organization through activation of PKCα and through phosphorylation P-PKCα (by phosphorylation of PKCα/beta (Thr638/641)). All the data elevated statistically and there is no statistically difference between control and DHEA-treated PCOS mouse group (P < 0.05).

**Figure 5:**
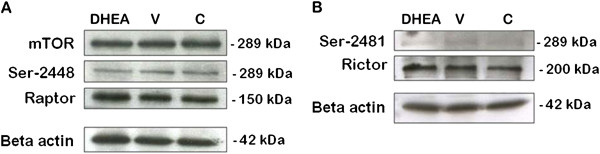
**mTORC1 and mTORC2 protein expression in control (C), DHEA-treated PCOS (DHEA) and vehicle (V) groups.** (P < 0.05).

**Figure 6:**
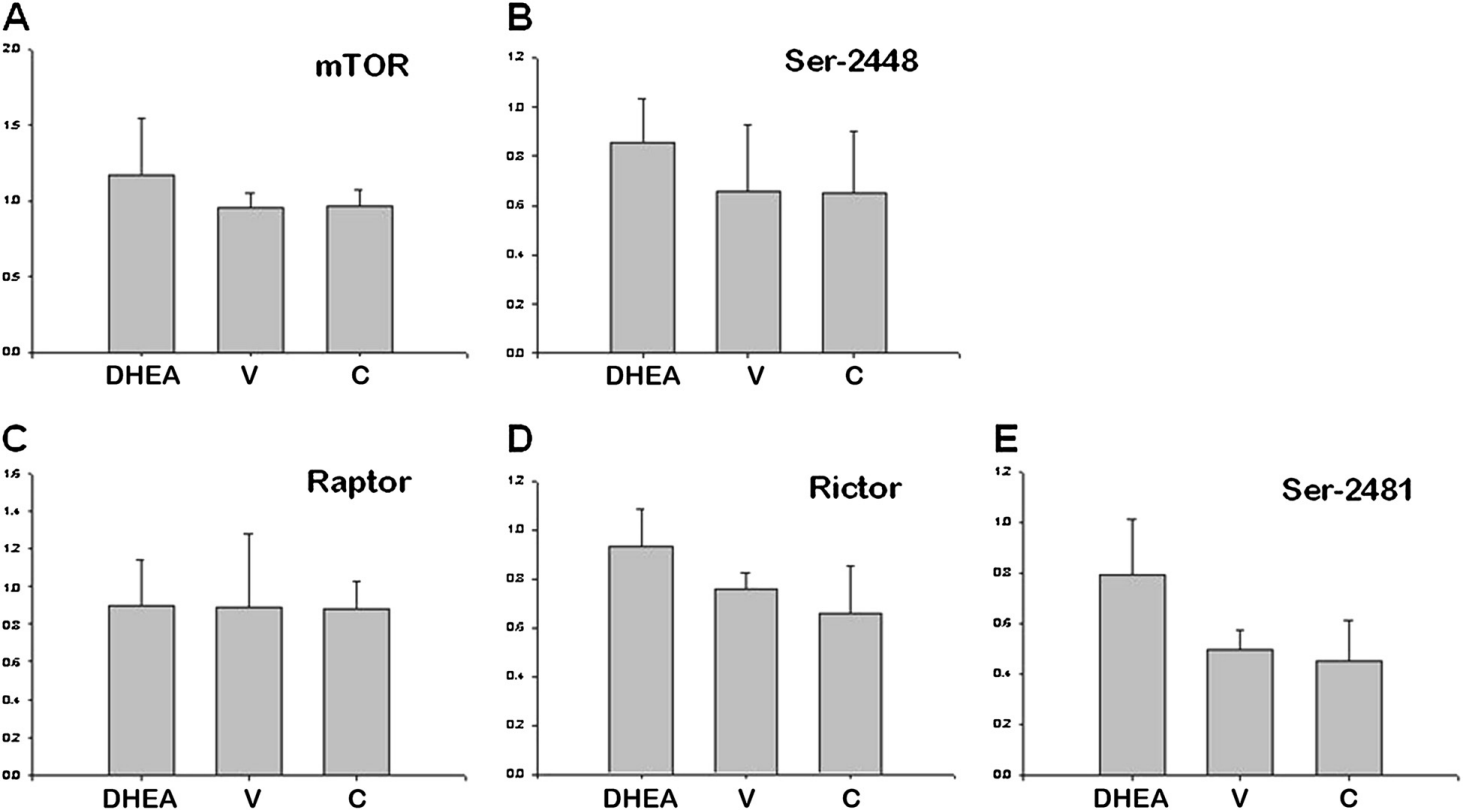
**mTORC1 and mTORC2 protein expression levels in control (C), DHEA-treated PCOS (DHEA) and vehicle (V) groups.** (P < 0.05).

mTOR signaling showed no difference between groups (Figures 5A and 6A). We found that P-mTOR (serine 2448) protein level was dramatically increased in PCOS group compared with that in control (Figures 5A and 6B). Raptor (Figures 5A and 6C) and GβL (LST8) (Figure 6A and B) protein signaling showed no difference between three groups (P < 0.05). mTORC2 is activated by phosphorylation of serine-2481 region of mTOR. P-mTOR (serine-2481) showed increased protein expression in PCOS group than other groups (Figures 5B and 6E). Rictor protein level was also higher in PCOS group as resemble with P-mTOR (serine-2481) (Figures 5B and 6D) (P < 0.05).

**Figure 7:**
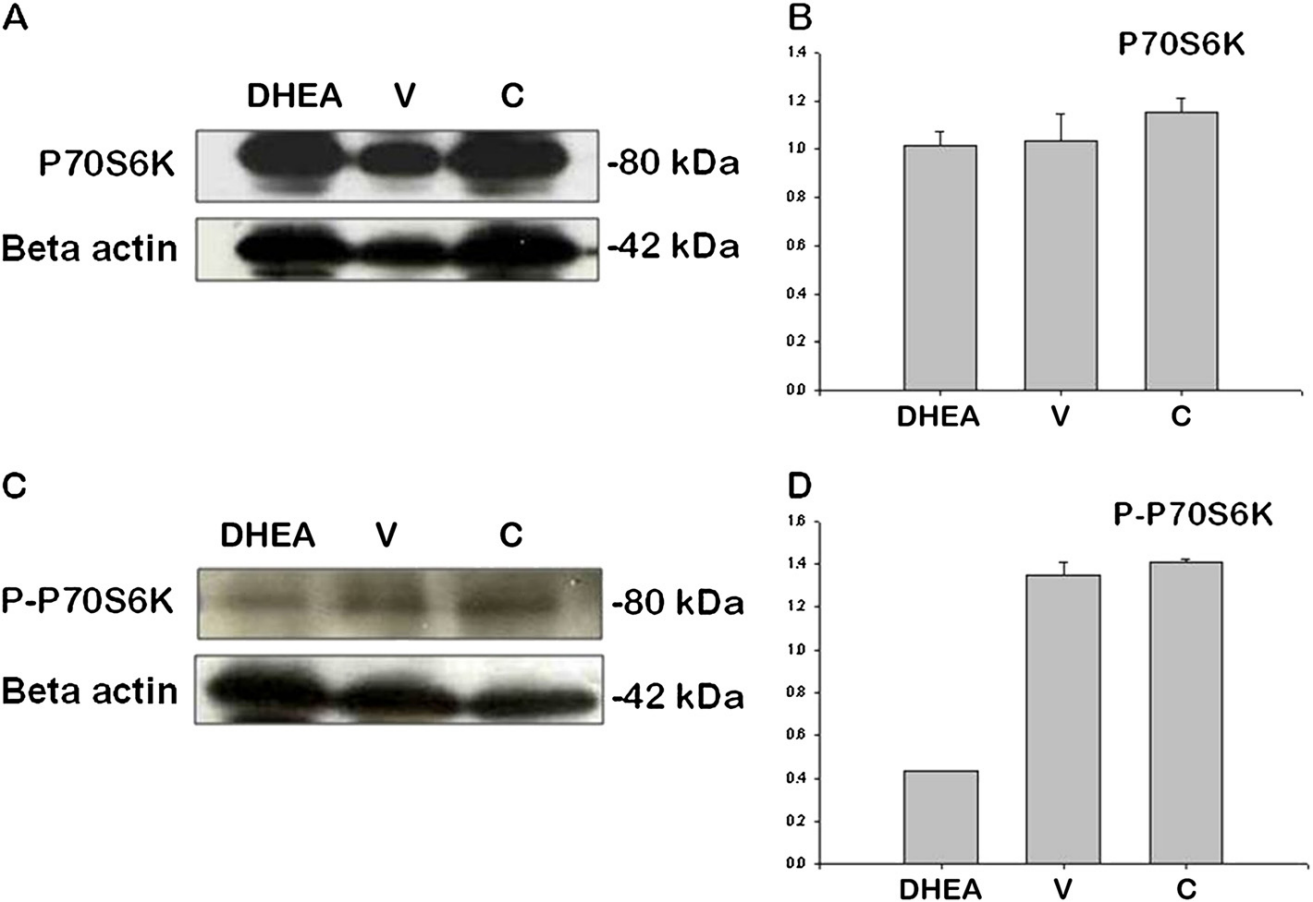
**P70S6K (A and B) and P-P70S6K (C and D) protein expression in control (C), DHEA-treated PCOS (DHEA) and vehicle (V) groups.** (P < 0.05).

**Figure 8:**
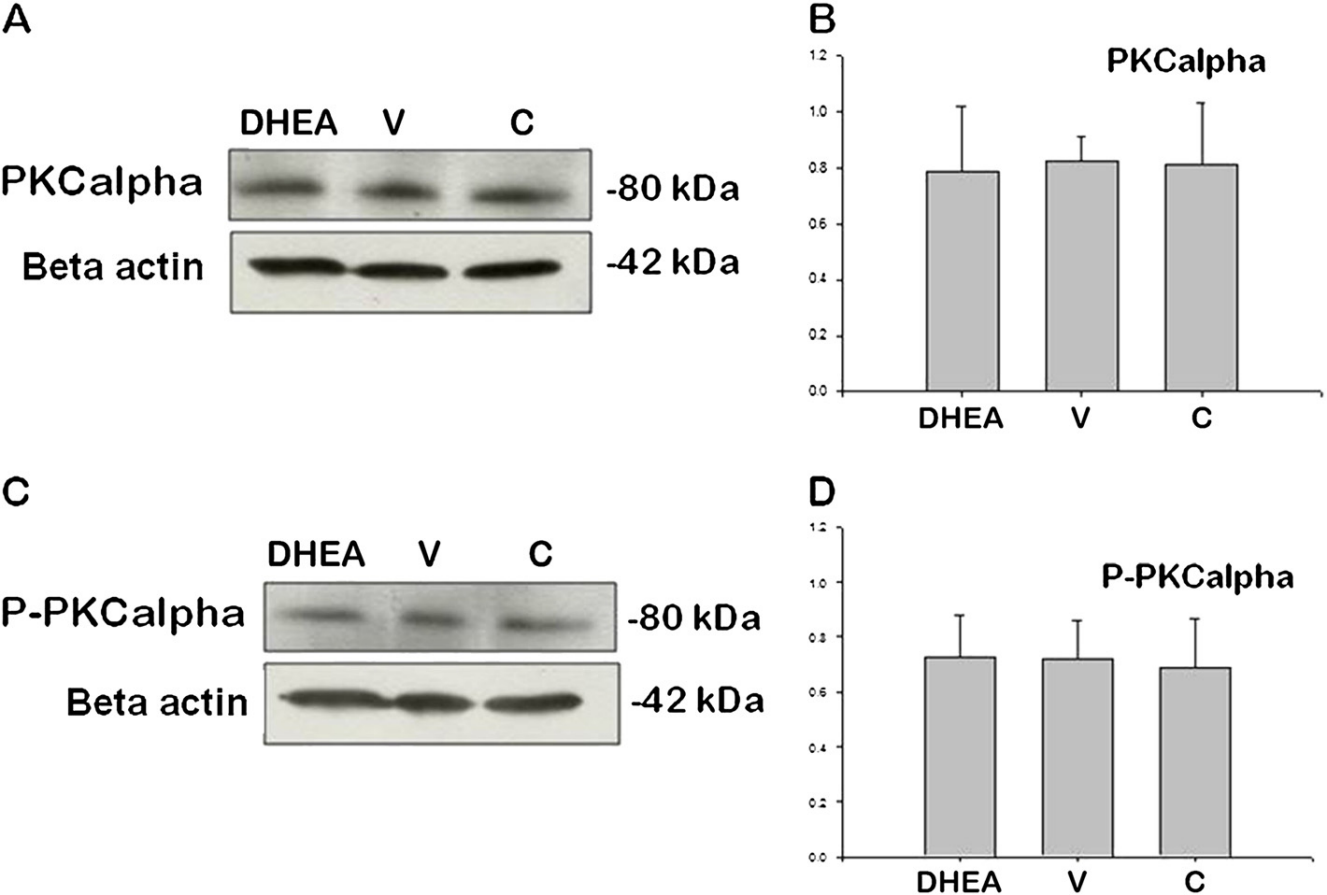
**PKCalpha (A and B) and P-PKCα/beta (Thr638/641 phosphorylation) (C and D) protein expression in control (C), DHEA-treated PCOS (DHEA) and vehicle (V) groups.** (P < 0.05).

**Figure 9:**
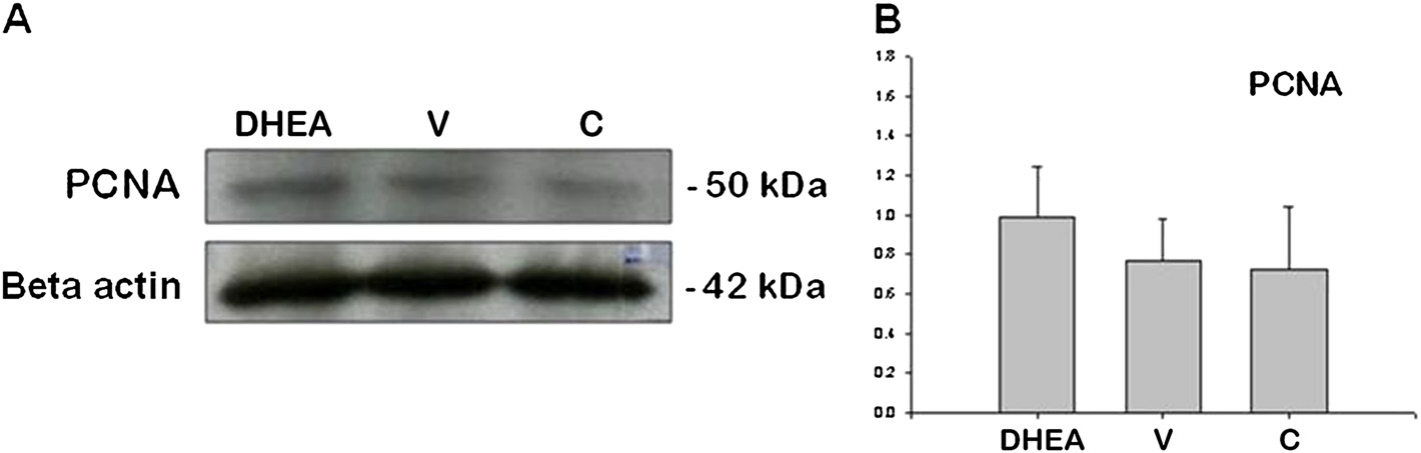
**PCNA protein expression in control (C), DHEA-treated PCOS (DHEA) and vehicle (V) groups.** (P < 0.05).

P70S6K protein band did not show any difference among three groups (Figure 7A and B) but mTORC1-dependent phosphorylation of P70S6K (P-P70S6K1) was increased in DHEA-treated group (Figure 7C and D) (P < 0.05). There were no difference of PKCα and P-PKCα/beta (Thr638/641 phosphorylation) signaling (Figure 8A–D) (P < 0.05). PCNA were used detection of proliferation difference in three groups by western blot. We showed that PCNA signaling was increased in PCOS group when we compared with control and vehicle groups (Figure 9A and B) (P < 0.05).

#### RT-PCR

DHEA-treated, vehicle and control group ovaries express the key components of both mammalian target of rapamycin complex 1 (mTORC1) and mTORC2. Reverse transcription polymerase chain reaction analysis of mTORC genes showed that the key components of both mTORC1 and mTORC2 are expressed in three groups of ovaries. Transcripts of 2 genes common to both complexes, mTOR and mLST8, are expressed in whole ovary samples (3 unique repeats, Ov1-3). Both Raptor, an essential component of mTORC1, and Rictor, essential for mTORC2, are expressed in whole ovary in all three groups. All mock reverse transcribed samples resulted in no bands. β-actin were used as internal control (Figure not shown).

## Discussion

In the present study we mimicked the human anovulatory PCOS using DHEA-treated mouse model. The population of antral follicles increased in PCOS mouse ovary, but there is evidence that disordered folliculogenesis also involves the smaller, preantral follicles [33]. Beside of increased number of growing immature follicle, communication in between oocyte and granulosa cells is disturbed. Therefore, there is a failure of selection of dominant follicle, and the number of arrested and atretic follicles is significantly increased in polycystic ovaries [34]. In our study, we observed excess immature and atretic follicles at the different stage of folliculogenesis. Ovarian morphology and hormone status were investigated in female rats given daily androstenedion injections and Okutsu et al. showed that androstenedione administration enhances apoptosis in the inner part of granulosa cell layers of antral follicles, which subsequently leads to the formation of ovarian follicular cysts and exposure to excess androstenedione stimulates premature luteinization of granulosa cells, which is most likely due to the loss of oocyte-granulosa cell communication [35]. The findings suggested here show that mice from the DHEA group exhibited increased levels of both serum estradiol (E2) and progesterone (P). We suggested that after daily injection of DHEA, hyperandrogenized environment occur the increased concentration of serum E2 would result in unfavorable conditions for producing follicles destined for ovulation. Previous studies work with the same animal model, have reported a similar hormonal regulation [16]. This hormonal profile suggests an increased steroidogenic activity, which is widely described in PCOS [1,36]. The follicles from anovulatory women with PCOS hypersecrete E2 when compared with size-matched follicles from normal ovaries or polycystic ovaries from ovulatory women [36-38]. Additionally, absent of corpora lutea structure in PCOS ovary showed an anovulatory infertility model.

A characteristic of the ovarian morphology that was seen in DHEA-treated mice was the presence of follicular cysts. These results demonstrate that ovarian follicular cysts are formed from antral follicles and that androstenedione treatments selectively disrupt the later stages of ovarian follicle development (follicle maturation) and subsequent ovulation. Thus, the current results demonstrate that enhanced E2 and P level is the cause of the follicular cyst formation, which may prevent the development of dominant follicles. Moreover, we showed that exposure to excess DHEA stimulated increased estradiol and progesterone levels. Therefore, excess DHEA stimulation may cause to the loss of oocyte-granulosa cell communication and degeneration of healthy oocytes and follicle [39,40]. Additionally, DHEA induces increased steroidogenesis in the proliferating and differentiative granulosa cells of early antral follicles as they develop into cysts.

In our present study, mTOR and its downstream effectors were elevated in DHEA-treated PCOS mouse model. The mTORC1 and mTORC2 have crucial roles in different pathways as energy and nutrient sensing, metabolism, cell growth, and differentiation [41-46]. We showed previously that mTOR is ubiquitously expressed in mouse ovary with predominantly cytoplasmic and perinuclear expression in granulosa cells. However, the P-mTOR (serine 2448) is strongly enriched within mitotic granulosa cells and localizes in the region of the mitotic spindle and also near actin filament–containing structures, including the contractile ring of cytokinesis [27]. Here we showed mTOR signal proteins in DHEA-treated PCOS mouse model. mTOR and P-mTOR (Serine-2448) showed more protein expression in P group than C and V group. Raptor and GβL (LST8) are two major components of mTORC1 have role in cell proliferation and differentiation. There is no difference about Raptor and GβL (LST8) expression between three groups. Therefore, we suggested that Raptor and GβL (LST8) proteins have important role in activation of mTORC1 and then stimulation of downstream proteins. In this situation, we suggested that Serine-2448 phosphorylated form of mTOR may be responsible from increased granulosa and theca cell proliferation in PCOS mouse ovary. Our PCNA western blot results confirmed that DHEA-treated PCOS mouse ovary showed increased proliferation compared to other groups as shown by increased PCNA expression. P70S6K is well defined downstream signal protein of mTORC1. mTOR regulates cell growth and proliferation functions through cytoplasmic targets such as P70S6K. Bachmann et al. showed that mTOR shuttles between the nucleus and cytoplasm, and nucleocytoplasmic shuttling of mTOR is required for the maximal activation of S6K1 [47]. mTOR stimulates translation by phosphorylating P70S6 kinase and, consequently, the 40S ribosomal protein S6 [48]. Activation of this pathway is required for FSH-mediated induction of several follicular differentiation markers, including luteinizing-hormone receptor (LHR), inhibin-α, microtubule-associated protein 2D, and the PKA type IIβ regulatory subunit [48]. Based on our results we suggested that activation of mTORC1 has stimulatory effect on P70S6K downstream signal protein. Therefore, P-P70S6K protein level showed difference than P70S6K. Interestingly in our study we detected decreased P-P70S6K protein expression in PCOS group and suggested that P-P70S6K might decrease in DHEA-treated PCOS mouse ovary because of dysfunctional folliculogenesis. Namely, follicular differentiation signals with FSH stimulation cannot show enough effect through decreased level of FSH in PCOS. Thence, we suggested that insufficient P70S6K activation causes the arrest of follicular development. Thus, P-P70S6K showed decreased level in DHEA-treated PCOS mouse ovary.

mTORC2 is composed of mTOR, Rictor, mSIN1, and GβL (LST8), and this branch of mTOR action is resistant to acute inhibition by rapamycin. Our study is the first to show showed weak P-mTOR (Serine-2481) protein level showed very weak expression in all groups, but increased in DHEA-treated PCOS mouse ovary. Moreover, Rictor protein level showed increased expression in PCOS mouse ovary when we compare with control and vehicle groups. We suggested that mTORC2 signal pathway may have important role in PCOS. PKCalpha is an important protein which has role in cell proliferation, differentiation and apoptosis. mTORC2 involved in the phosphorylation of PKCalpha and in post-translational processing [49]. Ablation of mTORC2 components (Rictor, Sin1 or mTOR) abolished phosphorylation of the PKCalpha [49]. In our study PKCalpha and its phosphorylated form P-PKCα/beta (Thr638/641) showed same intensity for protein level in three experimental groups. At the same time, there is no difference between PCOS and control group for PKCalpha and P-PKCα/beta (Thr638/641) protein levels. Therefore we suggested that mTORC2 may use also different downstream signal proteins beside PKCalpha in DHEA-treated PCOS mouse ovary. We therefore suggested that Rho and Akt downstream proteins of mTORC2 may also have roles in PCOS mouse ovary.

The characteristic morphological feature of polycystic ovaries in anovulatory women is accumulation of antral follicles in the range of 2–8 mm in diameter. Therefore follicular maturation is disturbed, resulting in premature arrest of follicular growth. Additionally oocyte-granulosa cells communication is also disrupted and apparent failure to select a dominant follicle [36]. The results presented here indicate that mTORC1 and mTORC2 together may have important regulatory function in PCOS mouse model. Different abnormalities may appear depending on stress or nutrition and they may effect directly follicular growth. As it is known that changes in lipid metabolism disorder in PCOS causes women to gain weight excessively. These findings in PCOS suggest a direct relationship between the energy metabolism of cells and the mTOR signaling mechanisms. Both stress and eating disorders have seen in PCOS (as commonly observed in patients with PCOS suffer from obesity) as well as hormonal imbalance. The mechanism of mTOR by affecting the development of cystic structures, and can cause anovulation. Therefore we suggested that the increase in mTOR activation (mTORC1 and mTORC2) causes increased granulosa cell proliferation. In another study, blockade of central mTOR signaling by rapamycin caused decreased LH secretion [26]. Consequently, we suggested that Rapamycin (the inhibitor of mTOR) may be a compensatory mechanism attempting to increase protein synthesis and regulate stimulation of luteinized hormone secretion for preventing or treating anovulatory PCOS.

PCOS patients have high levels of LH and show anovulatory follicles in their ovary. This might be due either to abnormalities in LH secretion or to an augmentation of the LH stimulus through hyperinsulinemia and/or hyperandrogenemia [50]. Phosphorylation of mTOR in PCOS mouse model may be potentially due to estrogens or other derivatives. So it is necessary to further investigate how DHEA is related, directly and indirectly mTOR signal mechanisms effect to folliculogenesis and ovulation process.

In summary, we have found that DHEA increases mTORC1 and mTORC2 expression in mouse ovary. It appears, since DHEA increased mTOR expression in proliferative and differentiative-stage cells (premature luteinization of granulosa cells), mTOR signal pathways in DHEA metabolism might play important roles in the PCOS mouse ovary that results in disturbance of the dominant follicle selection and leads to abnormal follicular development and cystogenesis.

## Competing interests

The authors declare that they have no competing interests.

## Authors' contributions

Both authors read and approved the final manuscript.
